# Supplementary material for: Early coordination of cell migration and cardiac fate determination during mammalian gastrulation
Source: EMBO J. 2025 May 13;44(12):3327–59. doi: 10.1038/s44318-025-00441-0 (PMC12170898; doi:10.1038/s44318-025-00441-0)
Supplement: Supplementary file 5 — Appendix [file 44318_2025_441_MOESM5_ESM.pdf]

Appendix for 'Early coordination of cell migration and cardiac fate determination during mammalian gastrulation'

Table of Contents

Appendix Fig. S1. **2**

Appendix Fig. S2. **3**

Appendix Fig. S3. **4**

Appendix Fig. S4. **6**

Appendix Fig. S5. **7**

Appendix Fig. S6. **8**

Appendix Fig. S7. **9**

Appendix Fig. S8. **10**

Appendix Fig. S9. **11**

Appendix Fig. S10. **12**

Appendix Fig. S11. **13**

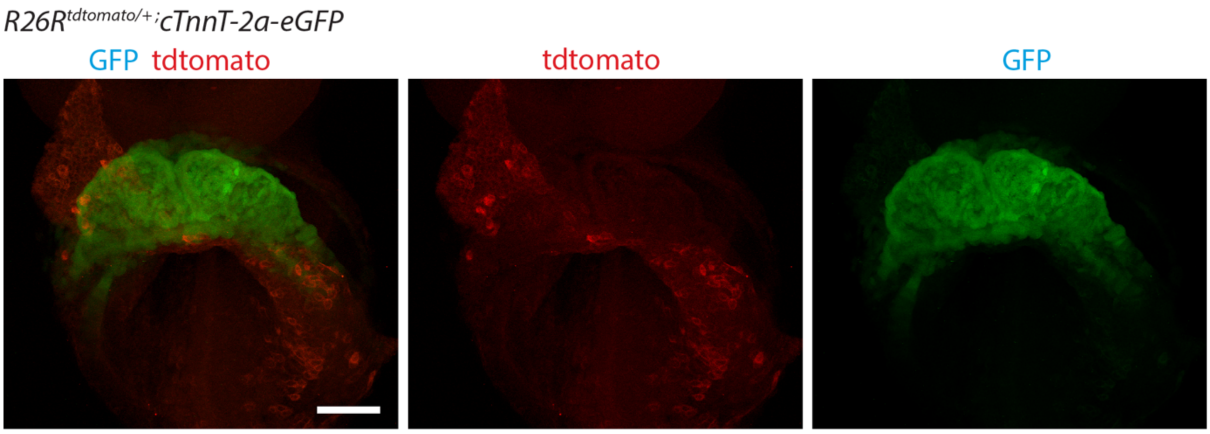

Appendix Fig. S1. Possible faint ectopic tdTomato expression in a R26Rtdtomato/+, cTnnT-2a-eGFP embryo in the absence of tamoxifen and cre induction.

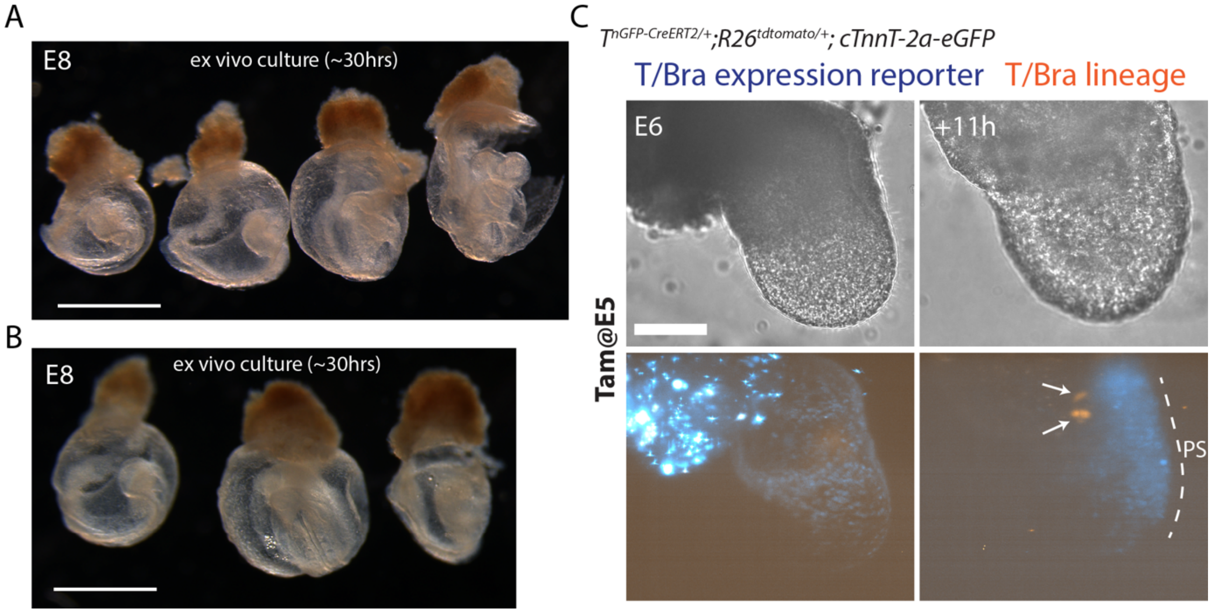

Appendix Fig. S2. Embryo Culture and Tamoxifen Administration.

(**A-B**) Two independent embryo culture. Scale bar: 1 mm (**C**) Time-lapse of $T^{nGPF\text{-}CreERT2/+}$; $R26R^{tdTomato/+}$; cTnnT-2a-eGFP embryos after tamoxifen administration (0.02 mg/body weight) at E5, followed by 11 hours of culture from E6.0 without tamoxifen. Arrows indicate tdTomato-positive cells in the extra-embryonic mesoderm. Scale bar: 100 µm. bw: body weight.

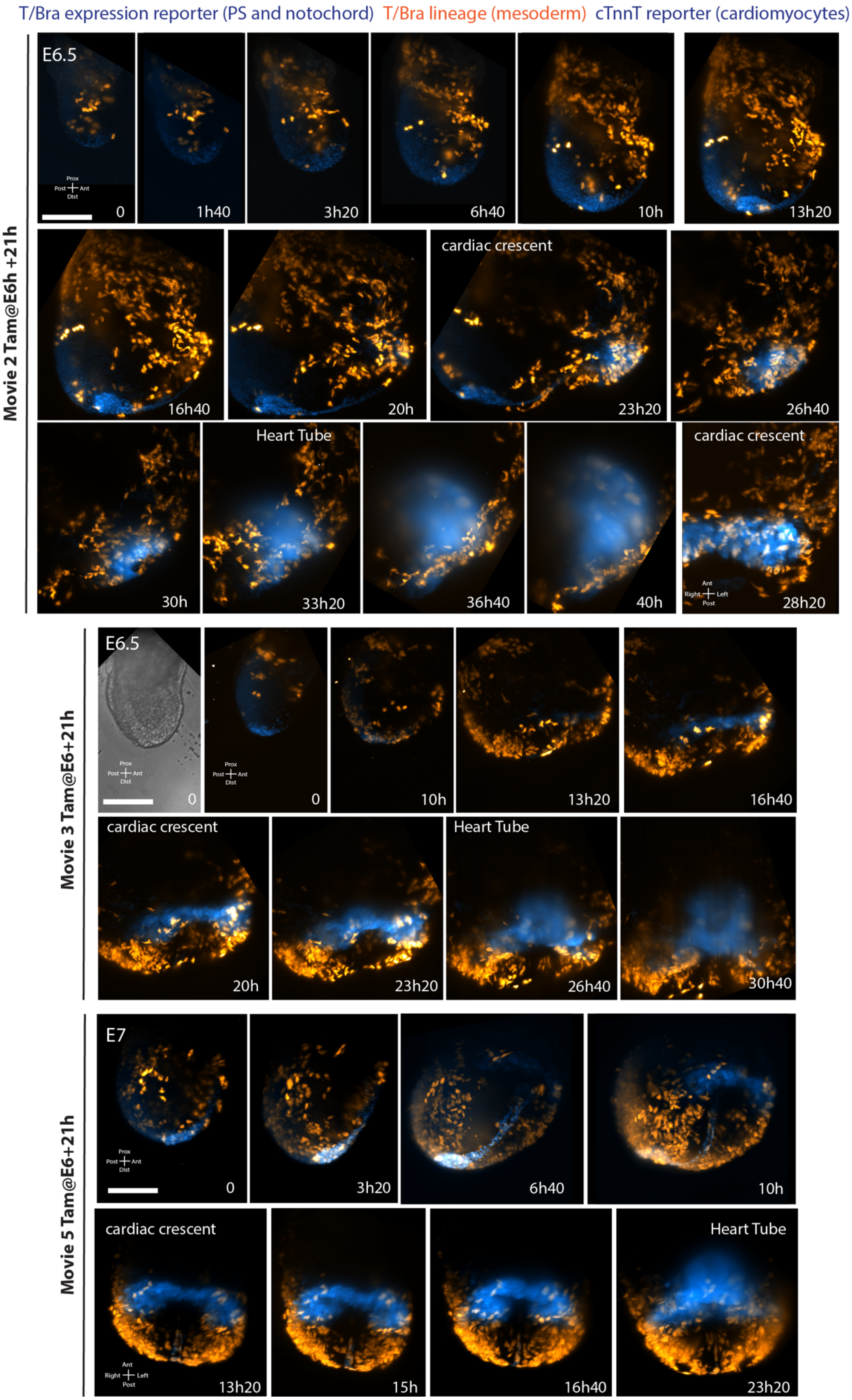

T/Bra expression reporter (PS and notochord) T/Bra lineage (mesoderm) cTnnT reporter (cardiomyocytes)
Movie 2 Tam@E6h +21h
E6.5
Prox
Post Ant
Dist
0
1h40
3h20
6h40
10h
13h20
16h40
20h
cardiac crescent
23h20
26h40
30h
Heart Tube
33h20
36h40
40h
cardiac crescent
Ant
Right Left
Post
28h20
Movie 3 Tam@E6+21h
E6.5
Prox
Post Ant
Dist
0
Prox
Post Ant
Dist
0
10h
13h20
16h40
cardiac crescent
20h
23h20
Heart Tube
26h40
30h40
Movie 5 Tam@E6+21h
E7
Prox
Post Ant
Dist
0
3h20
6h40
10h
cardiac crescent
Ant
Right Left
Post
13h20
15h
16h40
Heart Tube
23h20

Appendix Fig. S3. Long-term live-imaging from gastrulation to heart tube formation. Time-lapse sequences from $T^{nGPF\text{-}CreERT2/+}$;$R26R^{tdTomato/+}$; cTnnT-2a-eGFP embryos after tamoxifen administration (0.02 mg/body weight) at the indicated stage. LV/AVC indicates the left ventricle and atrioventricular canal. Images from A-movie 2 at 28:20 and movie 5 from 13:20 to 23:20 were computationally rotated in BigStitcher to show the ventral side. Scale bar: 100 μm. Ant: Anterior; Pos: Posterior.

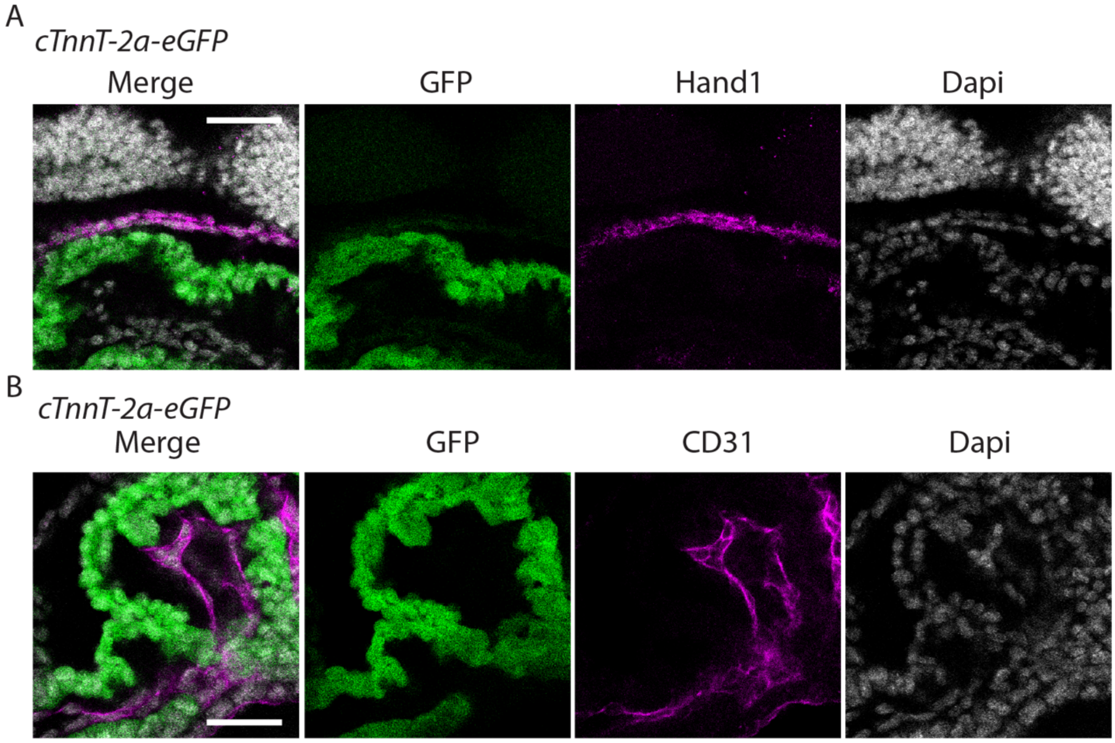

Appendix Fig. S4. At E8, the heart tube is composed of a Hand1^{+} pericardial outer layer and a CD31^{+} endocardial inner layer, surrounding the myocardium. (A) HCR labelling of an E8 cTnnT-2a-eGFP embryo revealing the Hand1^{+} pericardium. (B) Immunostaining of an E8 cTnnT-2a-eGFP embryo depicting the CD31^{+} endocardial cells. Scale bar: 50 µm**.**

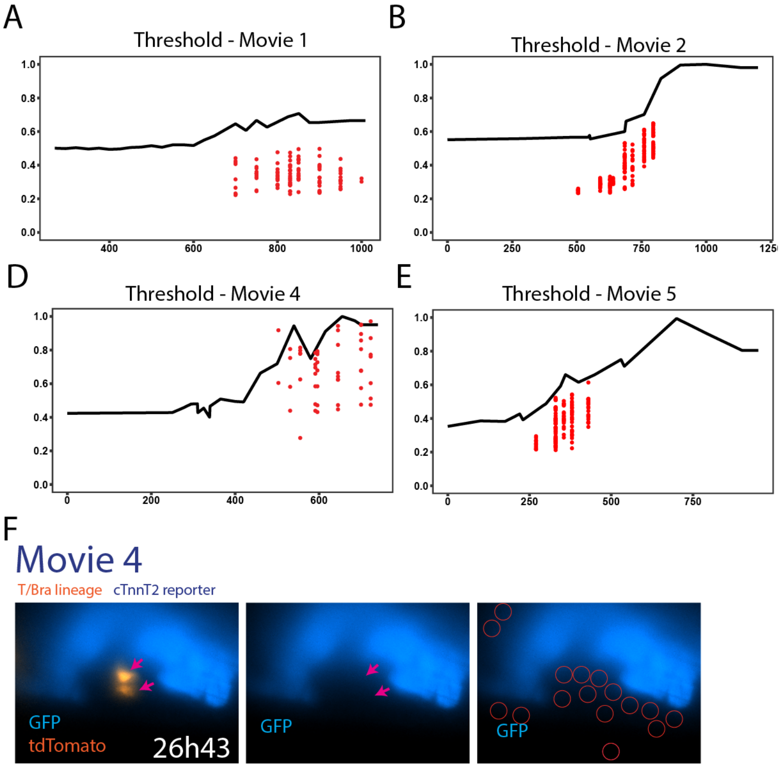

Appendix Fig. S5. Threshold Analysis

(**A-E**) Black lines denote the GFP intensity threshold separating GFP-positive cells from the background. Red points show GFP intensity values of endocardial cells in each movie. (**F**) Red circles indicate areas used to measure background GFP intensity.

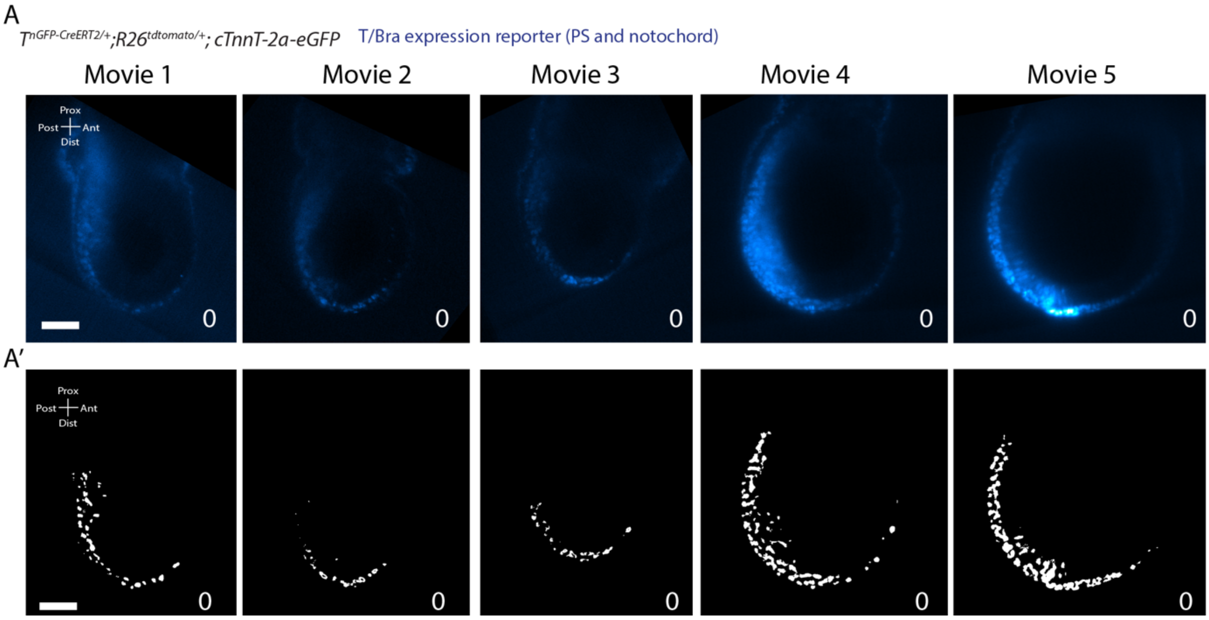

Appendix Fig. S6. nGFP signal intensity quantification.

((**A**) Single optical section of embryos at T0 from Movies 1 to 5, along with the corresponding segmentation of the primitive streak (**A'**). nGFP signal intensities were quantified within the segmented area.

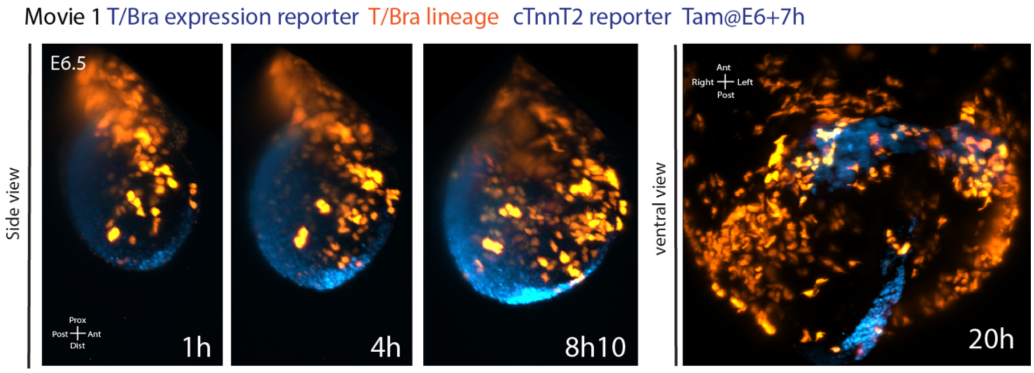

Appendix Fig. S7. Movie 1.

Images corresponding to embryo shown in Fig. 5A, with additional labeling to show T/Bra lineage-positive cells marked by tdTomato fluorescence.

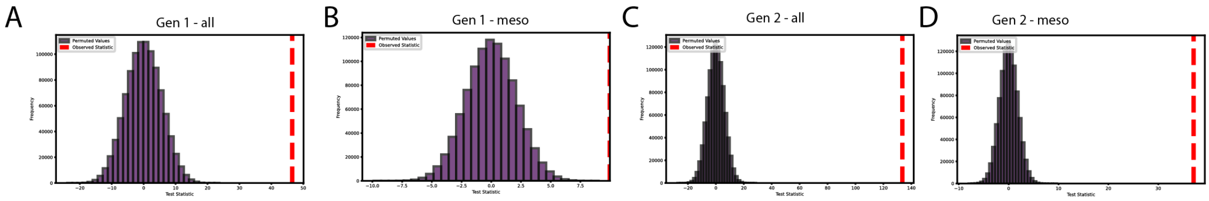

Appendix Fig. S8. Statistical tests

(A-D) Histograms showing the distribution of permuted tests based on the log mean difference between randomly shuffled uni-fated and bipotent DTW distances for: all D1 and D2 cells (A), D1 and D2 cells excluding ExEm progenitors (B), all D11, D12, D21, and D22 cells (C), and D11, D12, D21, and D22 cells excluding ExEm progenitors (D). Data are binned at 30 μm intervals, with the red dashed line indicating the observed log mean difference.

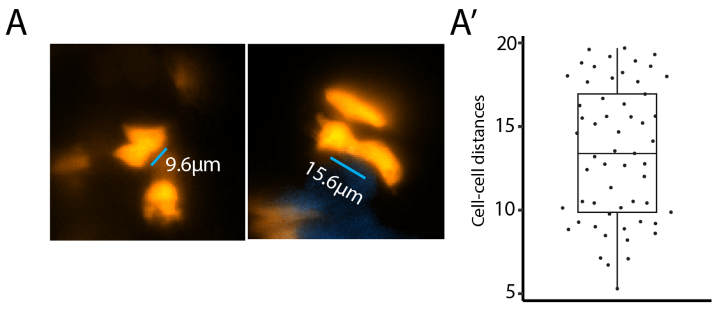

Appendix Fig. S9. Cell-cell contact measurements.

(A) Example of cell-cell distance measurement between cells that are in contact. (A') Mean cell-cell distances.

A

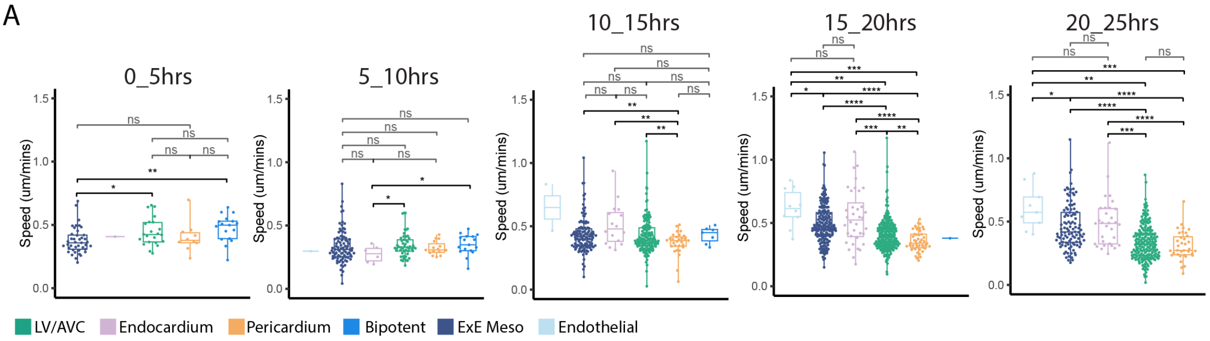

Appendix Fig. S10. Cell speed analysis.

(**A**) Cell speeds per cell fate were calculated across 5-hour time-periods. Movies were temporally aligned as shown in Fig. 5B. All statistical analyses were performed using the Mann-Whitney U test. LV/AVC: left ventricle and atrioventricular canal; ExEm: Extraembryonic mesoderm.

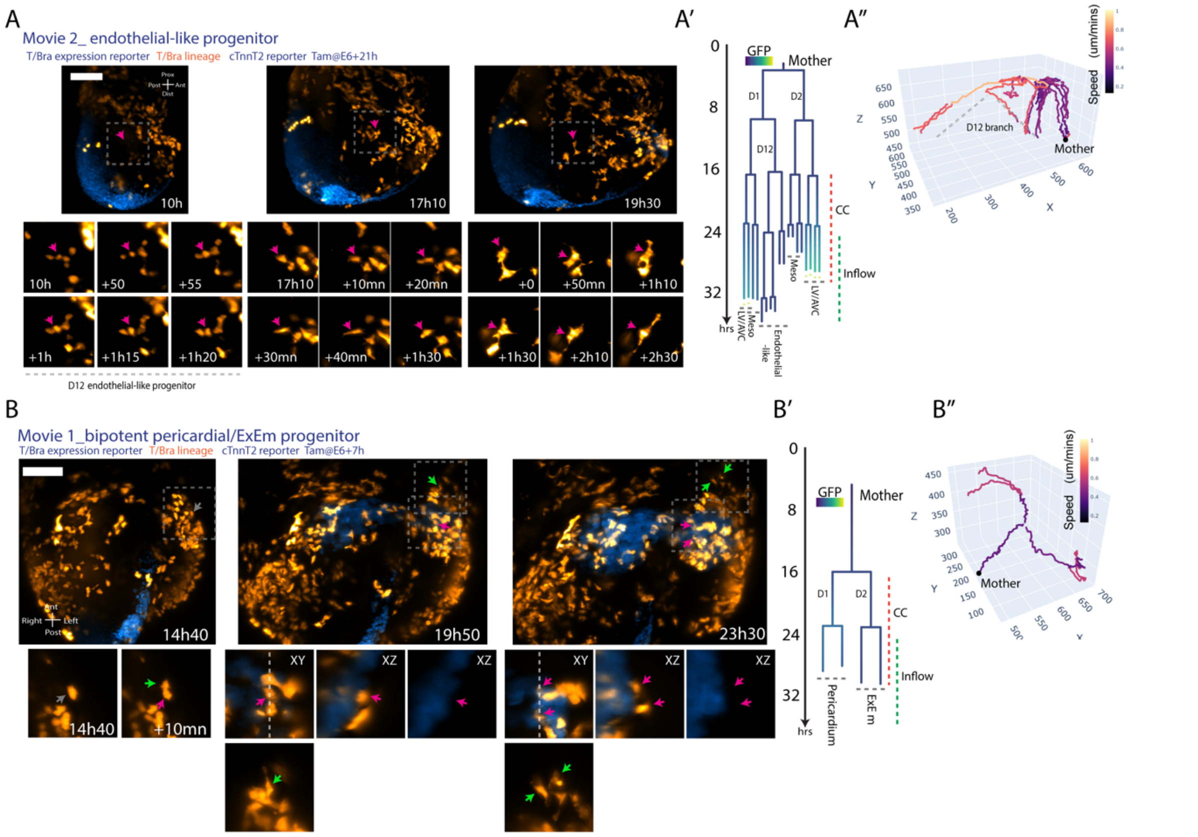

Appendix Fig. S11. Mesodermal cell behaviours

(A-B) Time-lapse images of *T*$^{nGPF\text{-}CreERT2/+}$*;R26R*$^{tdTomato/+}$*; cTnnT-2a-eGFP* embryos showing an endothelial-like progenitor (A) and a bipotent pericardial and ExEm progenitor (B). **(A)** Endothelial-like progenitor with progressive shape changes; lineage tree colored by normalized GFP intensity shown in (A'), with pink arrow indicating cell in branch D12. 3D plots (A'') color-coded by cell speed. **(B)** Bipotent pericardial and ExEm progenitor; lineage tree with normalized GFP intensity shown in (B'), with arrows indicating cells. 3D plot (B'') color-coded by cell speed. ExEm, extra-embryonic mesoderm; CC, cardiac crescent; Meso, mesoderm; LV/AVC, left ventricle-atrioventricular canal. Scale bar: 100 µm.
