# Supplementary figures and images for: Early coordination of cell migration and cardiac fate determination during mammalian gastrulation
Source: EMBO J. 2025 May 13;44(12):3327–59. doi: 10.1038/s44318-025-00441-0 (PMC12170898; doi:10.1038/s44318-025-00441-0)

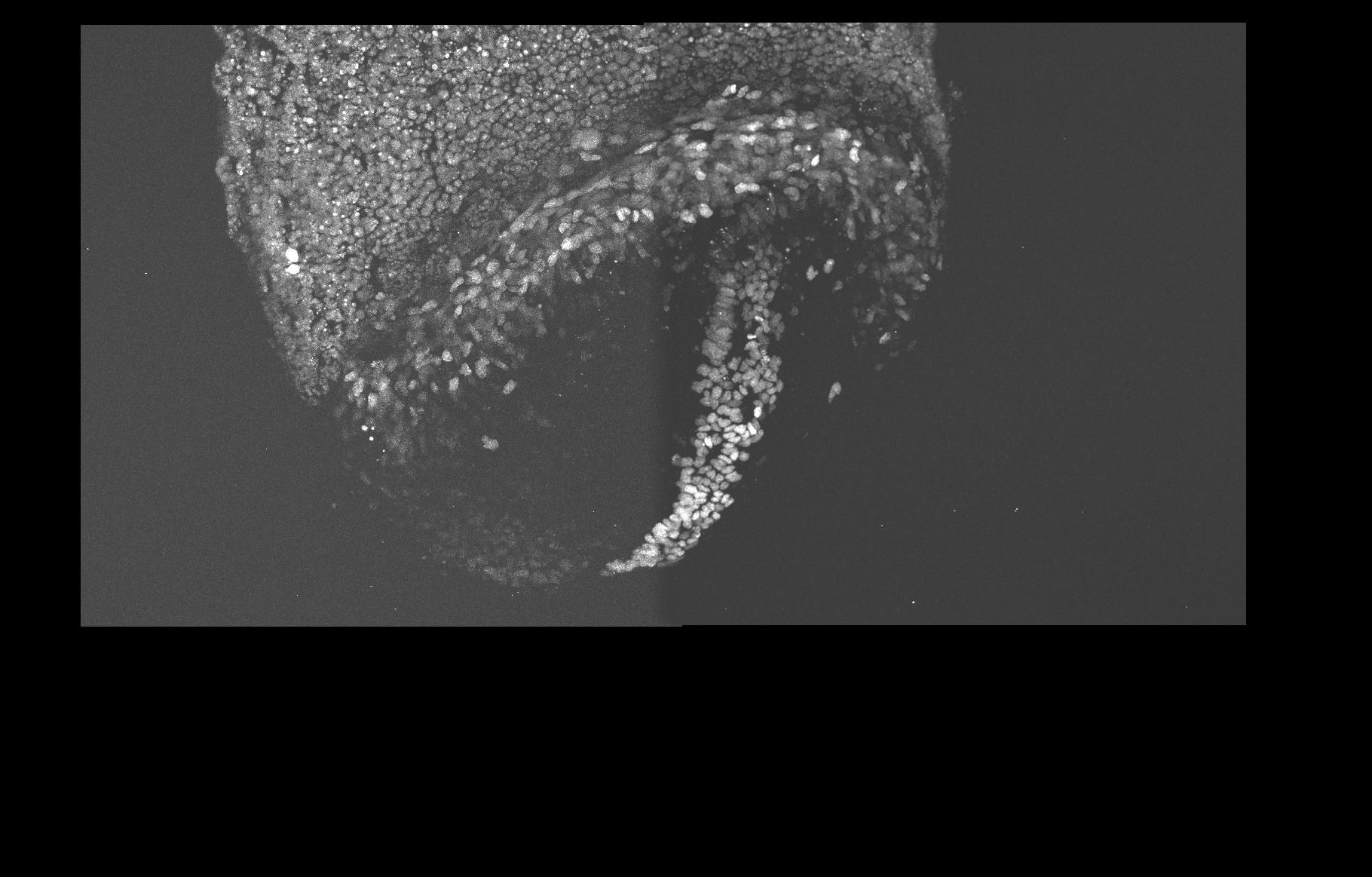

Supplement: Supplementary file 17 — Source data Fig. 1 [file 44318_2025_441_MOESM17_ESM.zip › Source Data Fig. 1/1D/Fig.1D.tif]

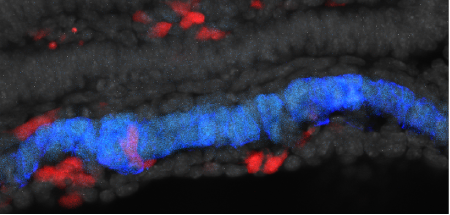

Supplement: Supplementary file 18 — Source data Fig. 2 [file 44318_2025_441_MOESM18_ESM.zip › Source Data Fig. 2/2C/2Ci.tif]

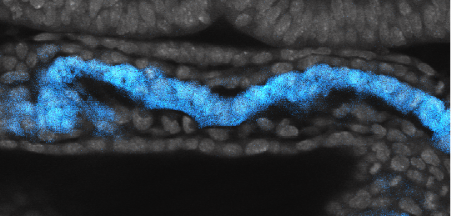

Supplement: Supplementary file 18 — Source data Fig. 2 [file 44318_2025_441_MOESM18_ESM.zip › Source Data Fig. 2/2C/2Civ.tif]

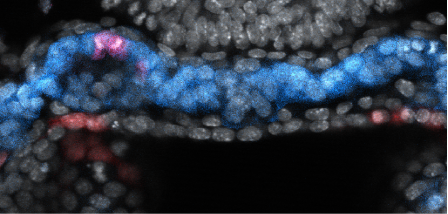

Supplement: Supplementary file 18 — Source data Fig. 2 [file 44318_2025_441_MOESM18_ESM.zip › Source Data Fig. 2/2C/2Ciii.tif]

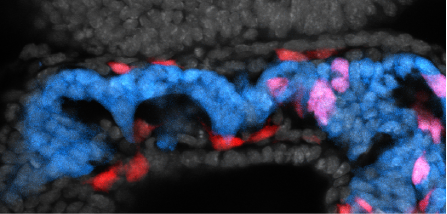

Supplement: Supplementary file 18 — Source data Fig. 2 [file 44318_2025_441_MOESM18_ESM.zip › Source Data Fig. 2/2C/2Cii.tif]

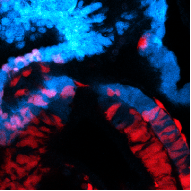

Supplement: Supplementary file 18 — Source data Fig. 2 [file 44318_2025_441_MOESM18_ESM.zip › Source Data Fig. 2/2E/2Ei_3.tif]

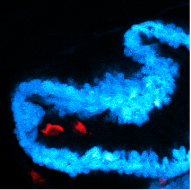

Supplement: Supplementary file 18 — Source data Fig. 2 [file 44318_2025_441_MOESM18_ESM.zip › Source Data Fig. 2/2E/2Ei_2.tif]

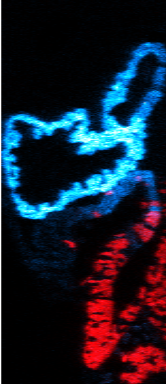

Supplement: Supplementary file 18 — Source data Fig. 2 [file 44318_2025_441_MOESM18_ESM.zip › Source Data Fig. 2/2E/2Eii_4.tif]

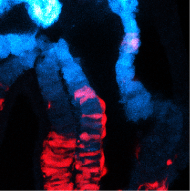

Supplement: Supplementary file 18 — Source data Fig. 2 [file 44318_2025_441_MOESM18_ESM.zip › Source Data Fig. 2/2E/2Eii_3.tif]

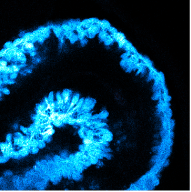

Supplement: Supplementary file 18 — Source data Fig. 2 [file 44318_2025_441_MOESM18_ESM.zip › Source Data Fig. 2/2E/2Eii_2.tif]

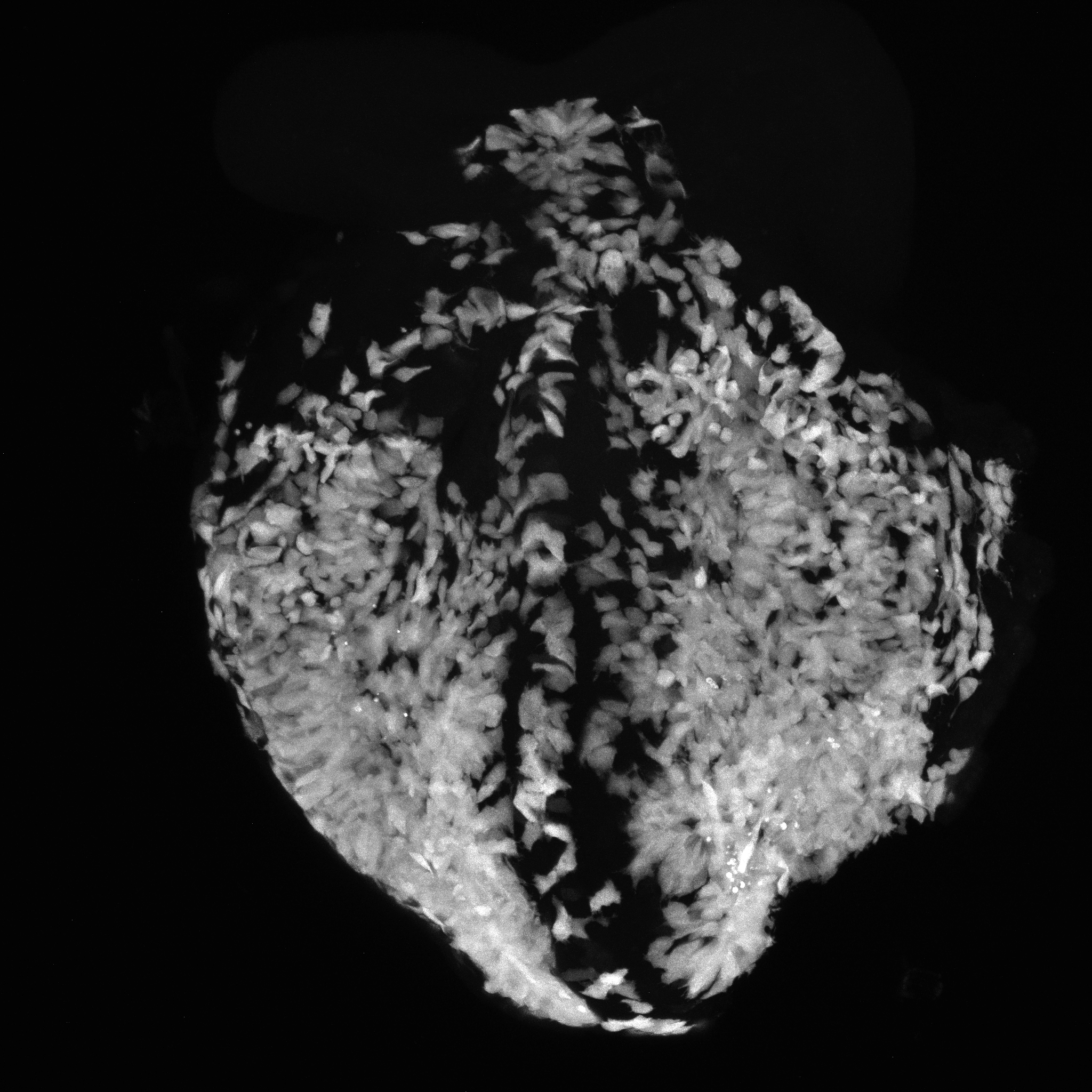

Supplement: Supplementary file 18 — Source data Fig. 2 [file 44318_2025_441_MOESM18_ESM.zip › Source Data Fig. 2/2B/2Bi.tif]

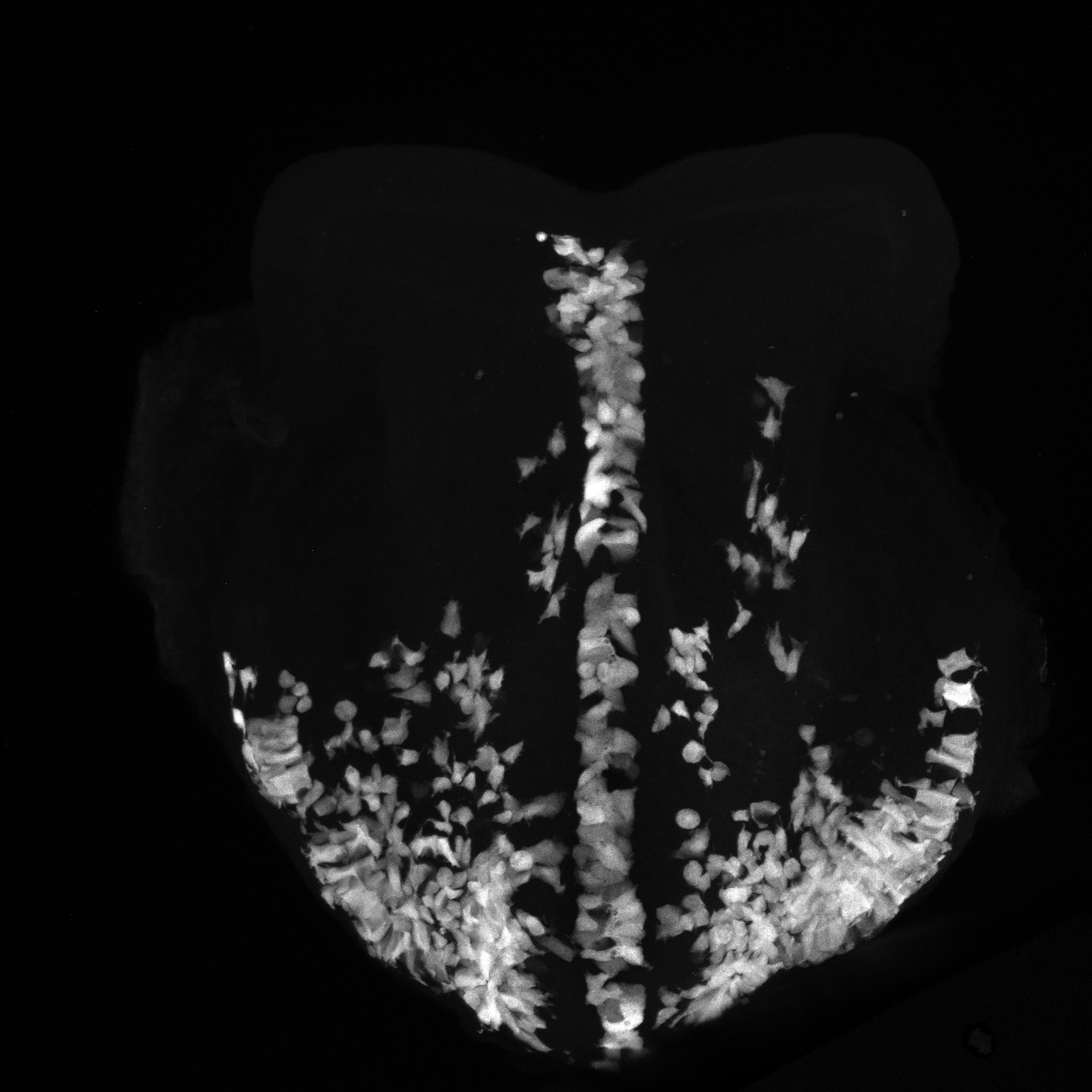

Supplement: Supplementary file 18 — Source data Fig. 2 [file 44318_2025_441_MOESM18_ESM.zip › Source Data Fig. 2/2B/2Biii.tif]

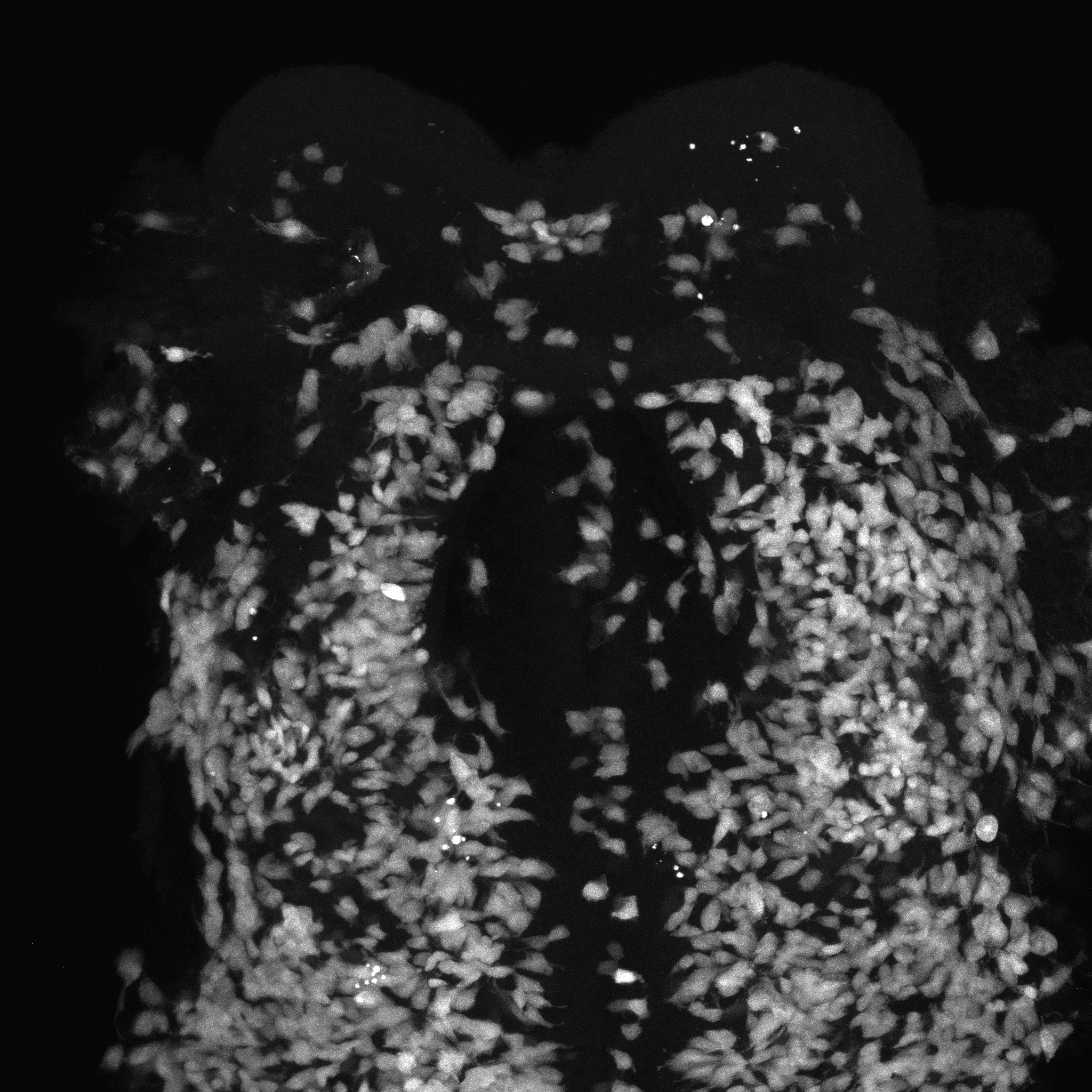

Supplement: Supplementary file 18 — Source data Fig. 2 [file 44318_2025_441_MOESM18_ESM.zip › Source Data Fig. 2/2B/2Bii.tif]

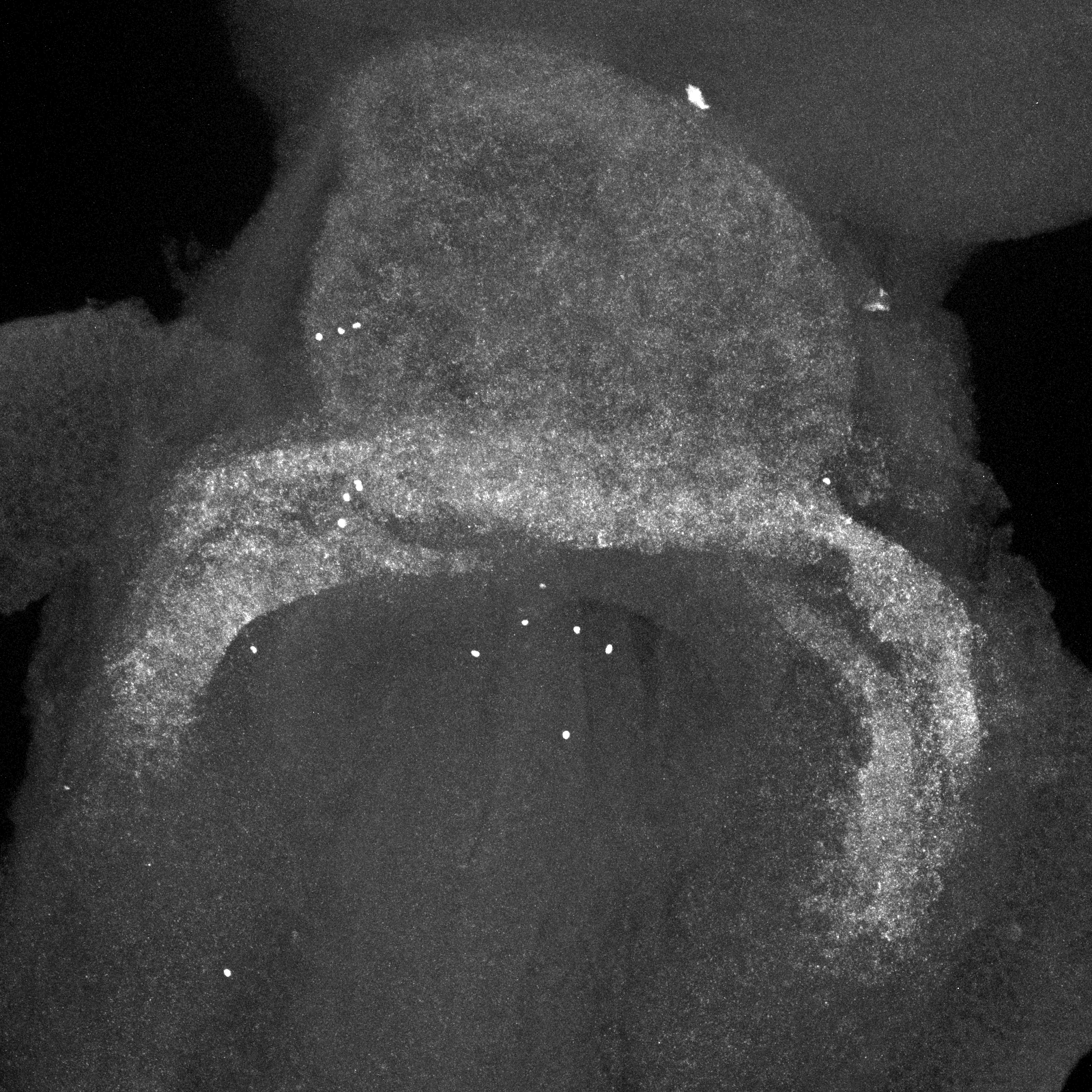

Supplement: Supplementary file 19 — Source data Fig. 3 [file 44318_2025_441_MOESM19_ESM.zip › Source Data Fig. 3/3E/3E_1.tif]

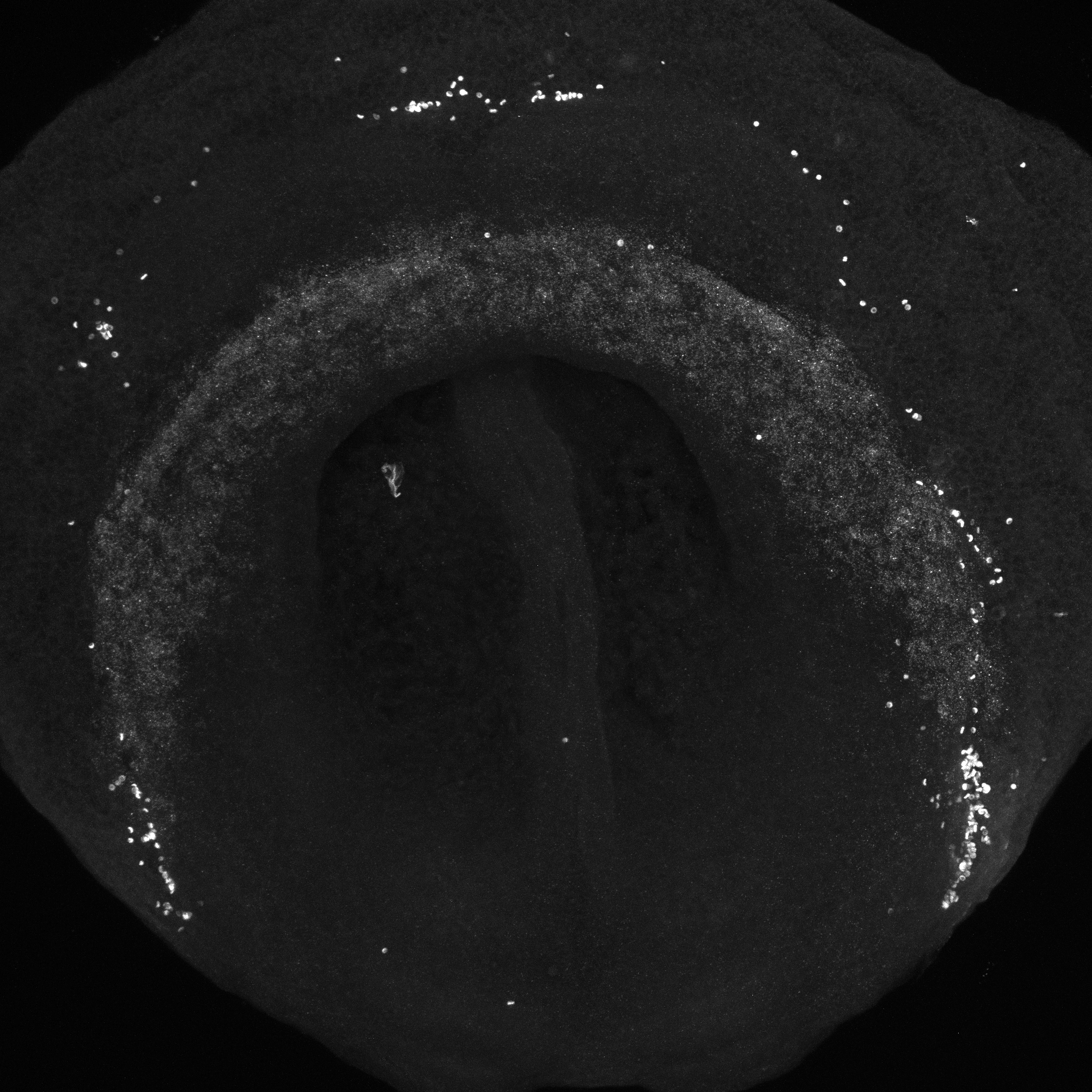

Supplement: Supplementary file 19 — Source data Fig. 3 [file 44318_2025_441_MOESM19_ESM.zip › Source Data Fig. 3/3E/3E_2.tif]

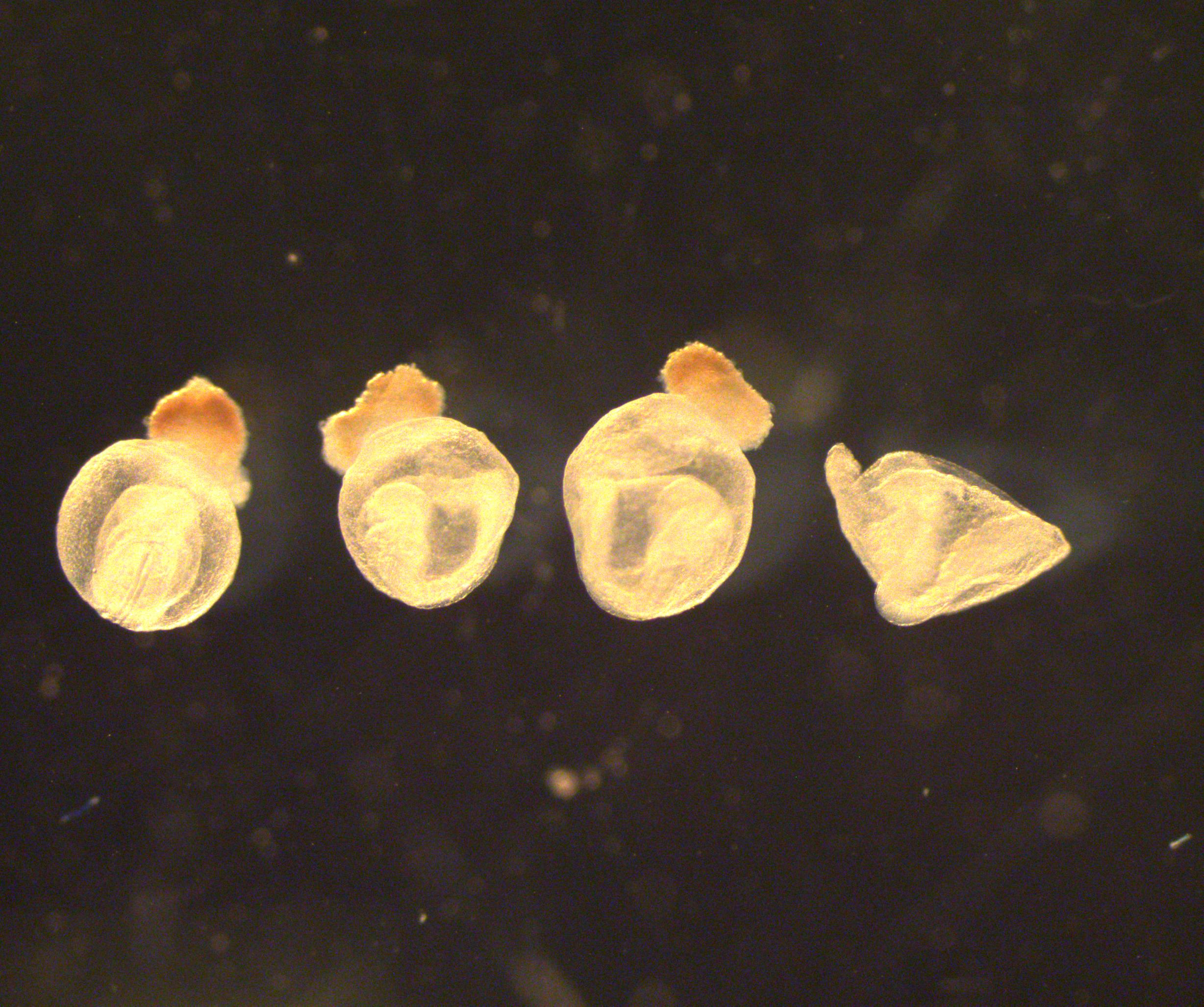

Supplement: Supplementary file 19 — Source data Fig. 3 [file 44318_2025_441_MOESM19_ESM.zip › Source Data Fig. 3/3B/3B.jpg]

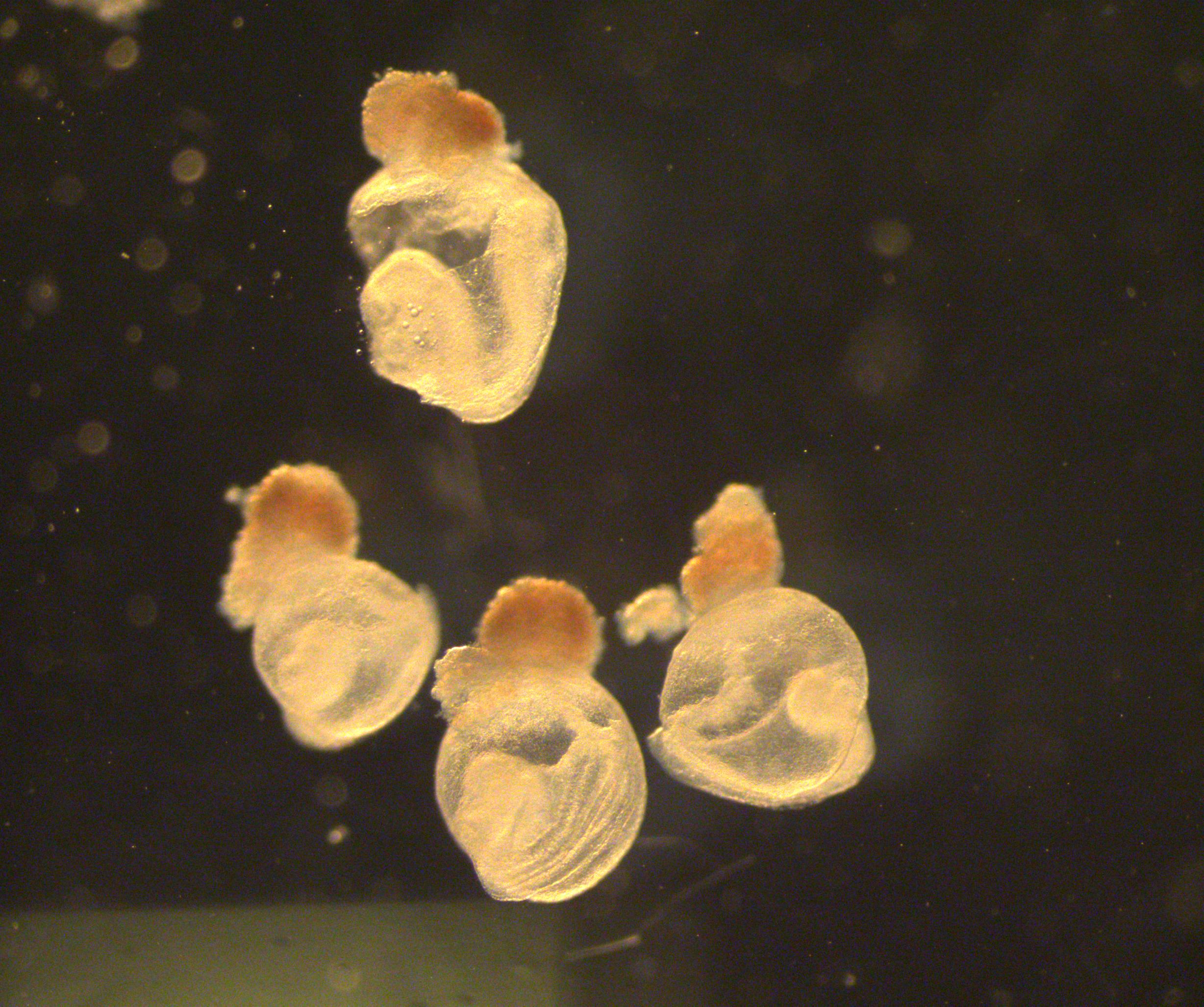

Supplement: Supplementary file 28 — EV and Appendix Figure Source Data [file 44318_2025_441_MOESM28_ESM.zip › Source Data for expanded views and appendix/Source Data Appendix Fig. S2/1A/1A.jpg]

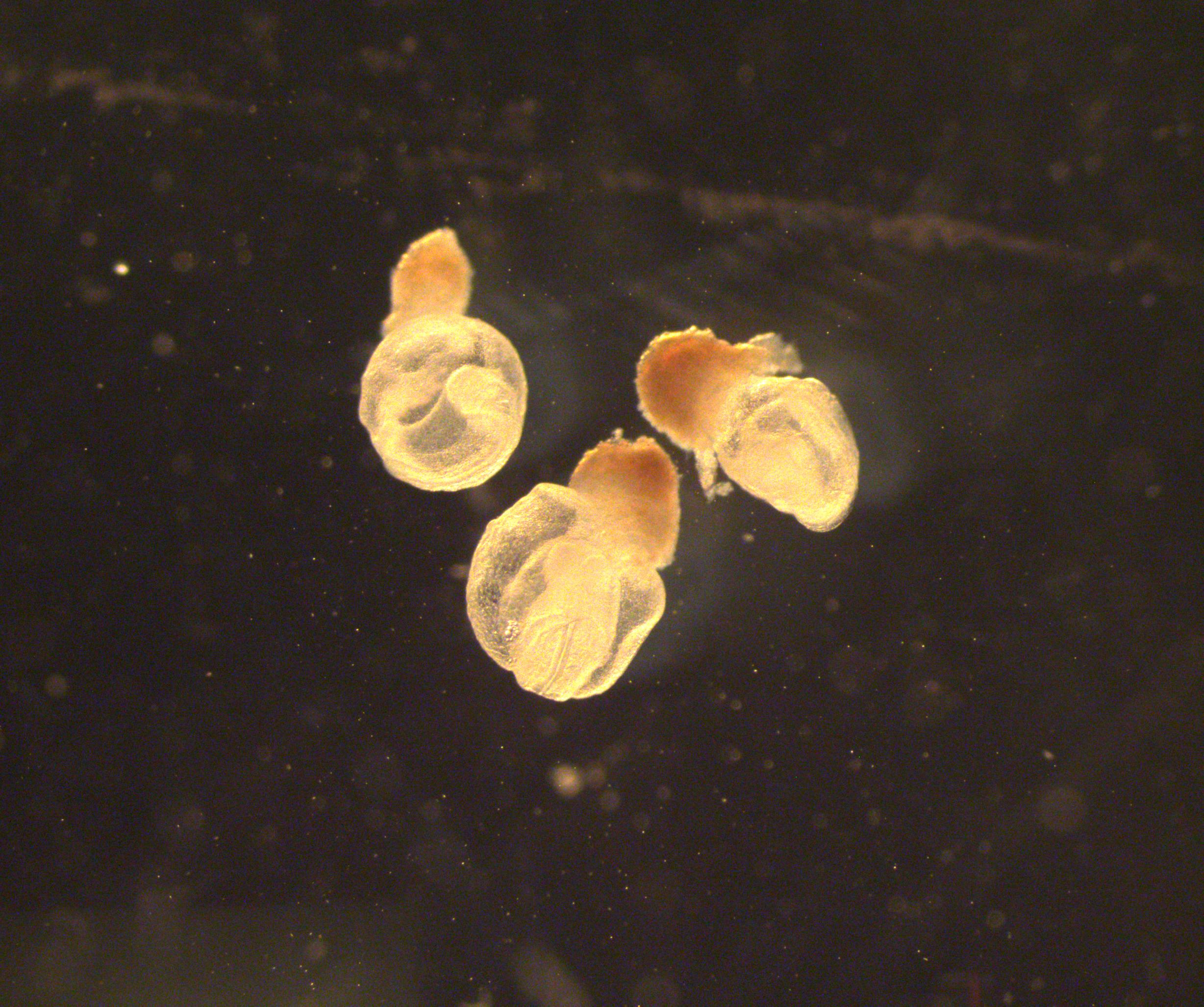

Supplement: Supplementary file 28 — EV and Appendix Figure Source Data [file 44318_2025_441_MOESM28_ESM.zip › Source Data for expanded views and appendix/Source Data Appendix Fig. S2/1B/1B.jpg]

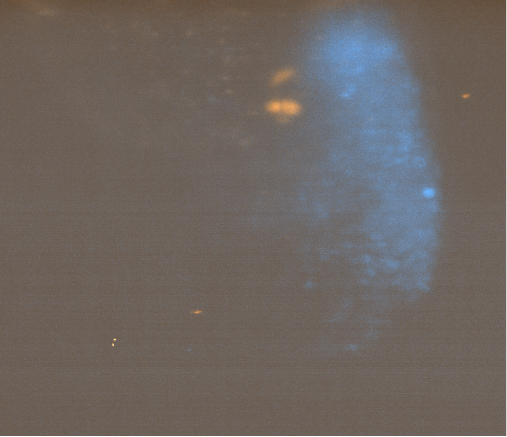

Supplement: Supplementary file 28 — EV and Appendix Figure Source Data [file 44318_2025_441_MOESM28_ESM.zip › Source Data for expanded views and appendix/Source Data Appendix Fig. S2/1C/1C_4.tif]

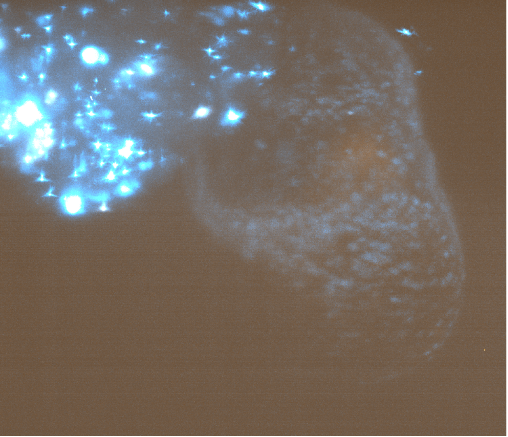

Supplement: Supplementary file 28 — EV and Appendix Figure Source Data [file 44318_2025_441_MOESM28_ESM.zip › Source Data for expanded views and appendix/Source Data Appendix Fig. S2/1C/1C_3.tif]

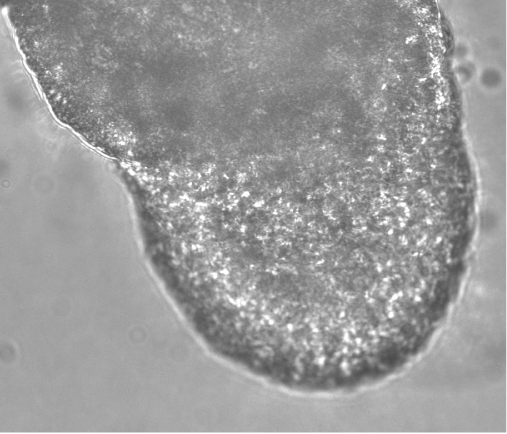

Supplement: Supplementary file 28 — EV and Appendix Figure Source Data [file 44318_2025_441_MOESM28_ESM.zip › Source Data for expanded views and appendix/Source Data Appendix Fig. S2/1C/1C_2.tif]

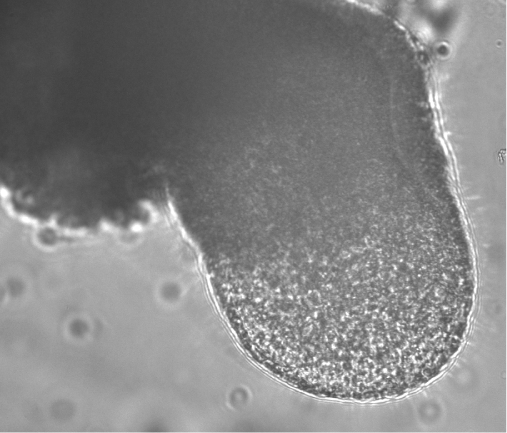

Supplement: Supplementary file 28 — EV and Appendix Figure Source Data [file 44318_2025_441_MOESM28_ESM.zip › Source Data for expanded views and appendix/Source Data Appendix Fig. S2/1C/1C_1.tif]
