# Supplementary material for: Early coordination of cell migration and cardiac fate determination during mammalian gastrulation
Source: EMBO J. 2025 May 13;44(12):3327–59. doi: 10.1038/s44318-025-00441-0 (PMC12170898; doi:10.1038/s44318-025-00441-0)
Supplement: Supplementary file 29 — Expanded View Figures [file 44318_2025_441_MOESM29_ESM.pdf]

Expanded View Figures

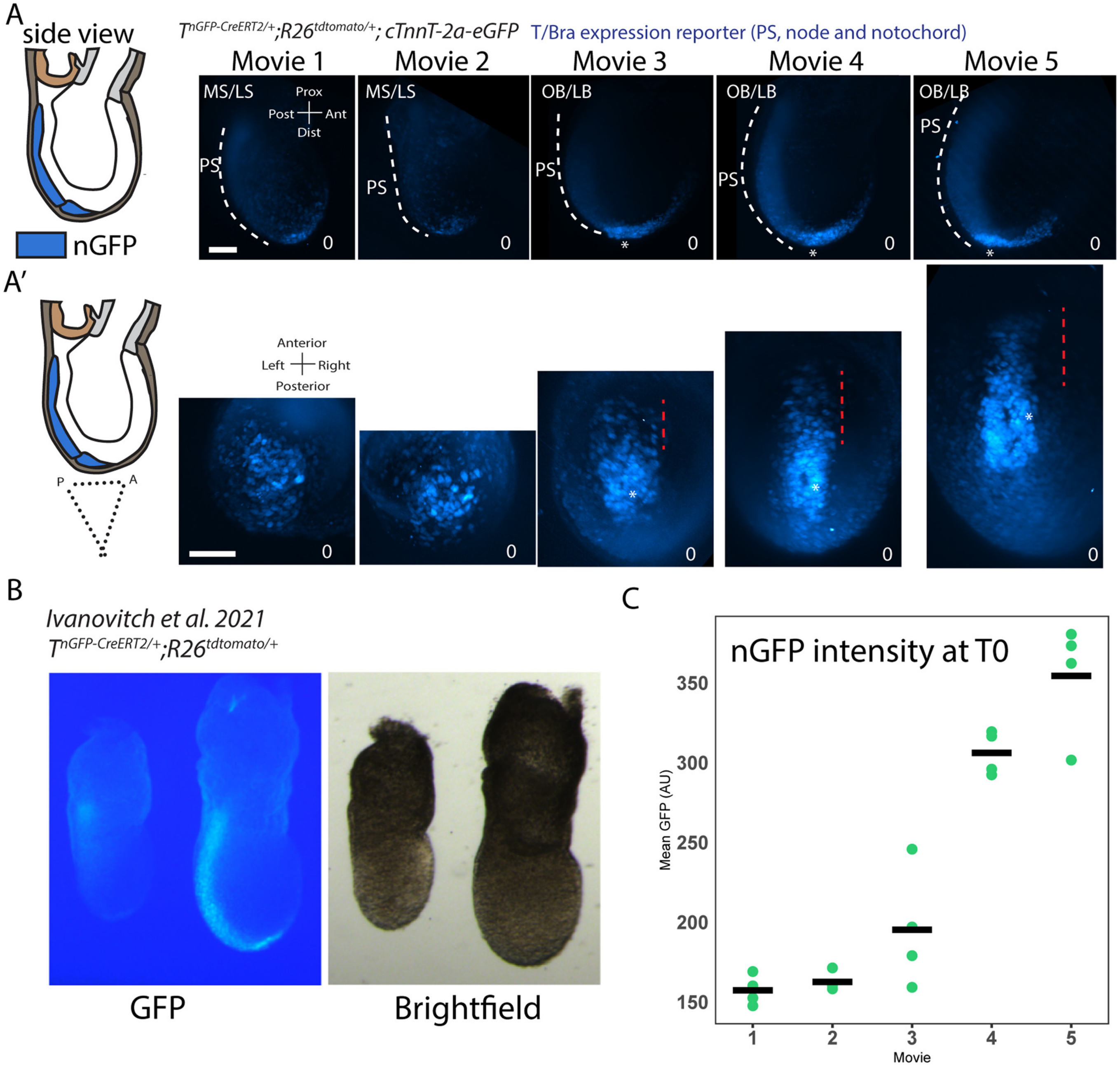

Figure EV1. Stage variation of the embryos imaged by light-sheet microscopy.

(**A–A′**) At T0, the embryos in Movies EV1 and 2 are at earlier stages compared to those in Movies 3 to 5. Embryos in Movies EV3–5 at T0 are characterized by the presence of a node structure (highlighted by an asterisk in (**A**)) and the beginning of the notochord plate (highlighted by the red dotted line in (**A′**)). In contrast, the node and notochord are absent in embryos from Movies EV1 and 2 at T0. (**B**) The earlier MS-LS embryos have a lower nGFP primitive streak signal compared to the later-stage OB-LB embryos. Images adapted from Ivanovitch et al, 2021. (**C**) Quantification of nGFP primitive streak signal at T0 for embryos from Movies EV1–5. nGFP was quantified in four consecutive optical sections (see also Appendix Fig. S6). Each data point represents the mean GFP intensity for each z-slices. PS: primitive streak. Scale bar: 200 µm in (**A**) and 100 µm in (**A′**). MS-LS: mid and late streak stages. OB/LB: no bud to late bud stages. nGFP: nuclear GFP. Source data are available online for this figure.

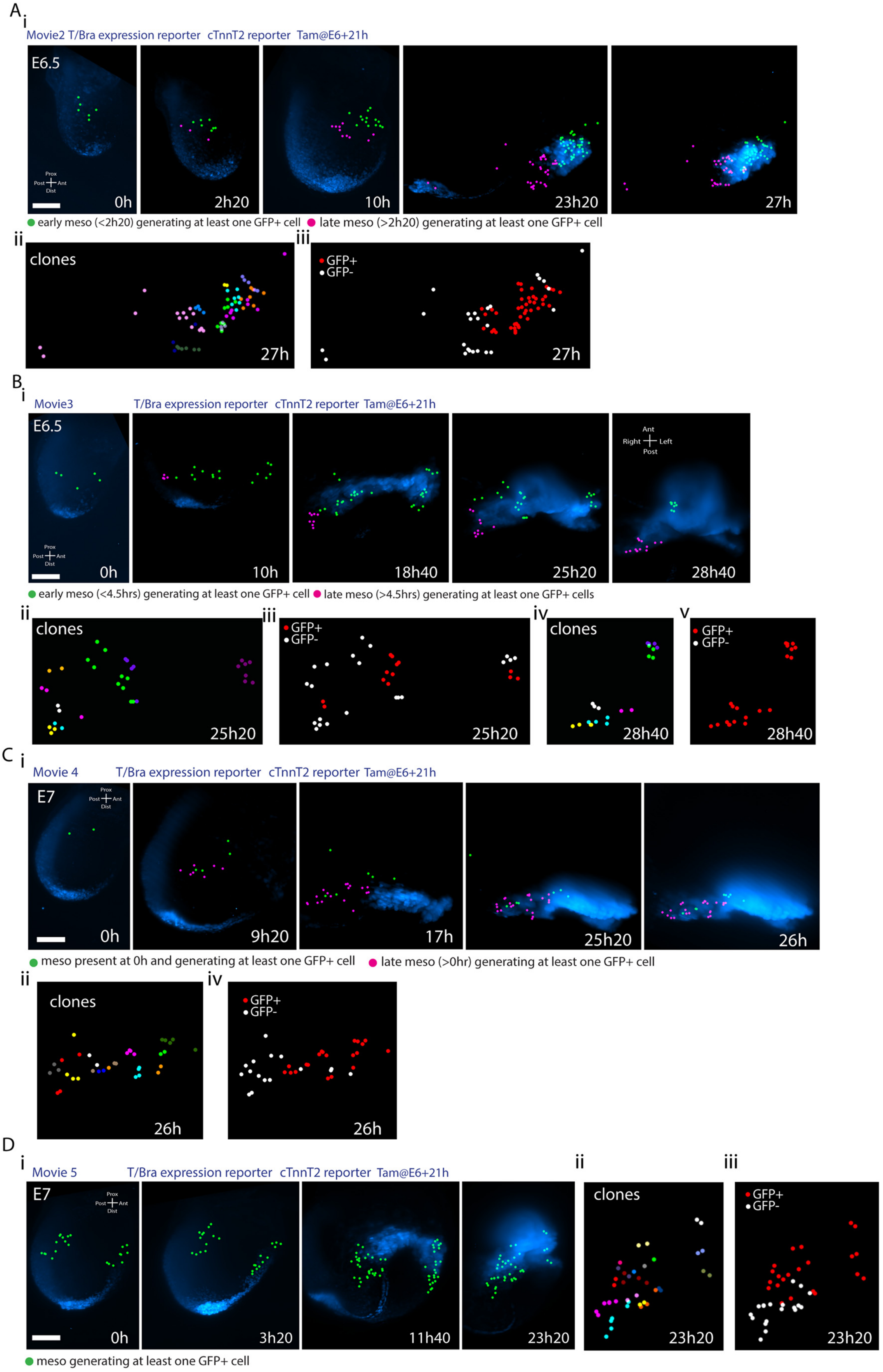
A
i
Movie2 T/Bra expression reporter cTnnT2 reporter Tam@E6+21h
E6.5
0h
2h20
10h
23h20
27h
early meso (<2h20) generating at least one GFP+ cell
late meso (>2h20) generating at least one GFP+ cell
ii
clones
27h
iii
GFP+
GFP-
27h
B
i
Movie3
T/Bra expression reporter cTnnT2 reporter Tam@E6+21h
E6.5
0h
10h
18h40
25h20
28h40
early meso (<4.5hrs) generating at least one GFP+ cell
late meso (>4.5hrs) generating at least one GFP+ cells
ii
clones
25h20
iii
GFP+
GFP-
25h20
iv
clones
28h40
v
GFP+
GFP-
28h40
C
i
Movie 4
T/Bra expression reporter cTnnT2 reporter Tam@E6+21h
E7
0h
9h20
17h
25h20
26h
meso present at 0h and generating at least one GFP+ cell
late meso (>0hr) generating at least one GFP+ cell
ii
clones
26h
iv
GFP+
GFP-
26h
D
i
Movie 5
T/Bra expression reporter cTnnT2 reporter Tam@E6+21h
E7
0h
3h20
11h40
23h20
meso generating at least one GFP+ cell
ii
clones
23h20
iii
GFP+
GFP-
23h20

 © The Author(s)

Figure EV2. Independent progenitors contribute to the LV/AVC and Atria.

(A–D) Only progenitors contributing at least one cTnnT-2a-GFP+ cell are displayed. (i) Fate map showing early (green) and late (magenta) mesoderm contributions to heart tube regions (early/late classification is arbitrary). (ii) Each colour represents a unique clone. (iii) cTnnT-2a-GFP+ cells in red. Scale bar: 100 µm.

© The Author(s)

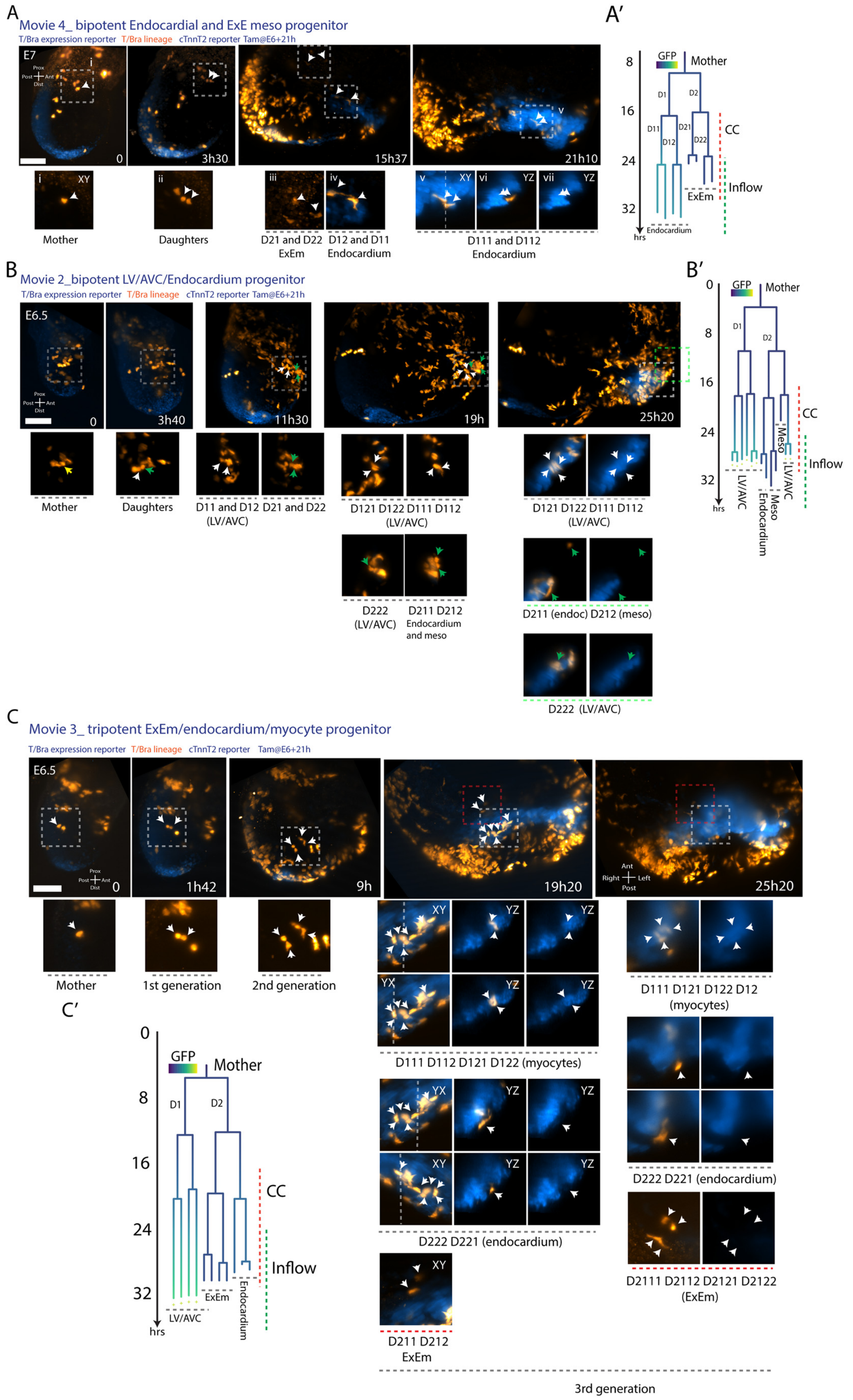
A
Movie 4_ bipotent Endocardial and ExE meso progenitor
T/Bra expression reporter T/Bra lineage cTnnT2 reporter Tam@E6+21h
E7
0
3h30
15h37
21h10
Mother
Daughters
D21 and D22 ExEm
D12 and D11 Endocardium
D111 and D112 Endocardium
A′
GFP
Mother
CC
Inflow
ExEm
Endocardium
hrs
B
Movie 2_bipotent LV/AVC/Endocardium progenitor
T/Bra expression reporter T/Bra lineage cTnnT2 reporter Tam@E6+21h
E6.5
3h40
11h30
19h
25h20
Mother
Daughters
D11 and D12 (LV/AVC)
D21 and D22
D121 D122 D111 D112 (LV/AVC)
D121 D122 D111 D112 (LV/AVC)
D222 (LV/AVC)
D211 D212 Endocardium and meso
D211 (endoc) D212 (meso)
D222 (LV/AVC)
B′
LV/AVC
Endocardium
Meso
C
Movie 3_ tripotent ExEm/endocardium/myocyte progenitor
T/Bra expression reporter T/Bra lineage cTnnT2 reporter Tam@E6+21h
E6.5
1h42
9h
19h20
25h20
Mother
1st generation
2nd generation
D111 D112 D121 D122 (myocytes)
D222 D221 (endocardium)
D211 D212 ExEm
D111 D121 D122 D12 (myocytes)
D222 D221 (endocardium)
D2111 D2112 D2121 D2122 (ExEm)
3rd generation
C′
GFP
Mother
CC
Inflow
LV/AVC
ExEm
Endocardium
hrs

 © The Author(s)

Figure EV3. Examples of bipotent and tripotent mesodermal progenitors.

(**A–C**) Time-lapse images of *T*$^{nGPF\text{-}CreERT2/+}$;*R26R*$^{tdTomato/+}$; *cTnnT-2a-eGFP* embryos showing bipotent (**A**, **B**) and tripotent (**C**) progenitors. Corresponding lineage trees, coloured by normalized GFP intensity, are shown in (**A′–C′**), with arrows indicating the cells in (**A–C**). Scale bar: 100 μm. Source data are available online for this figure.

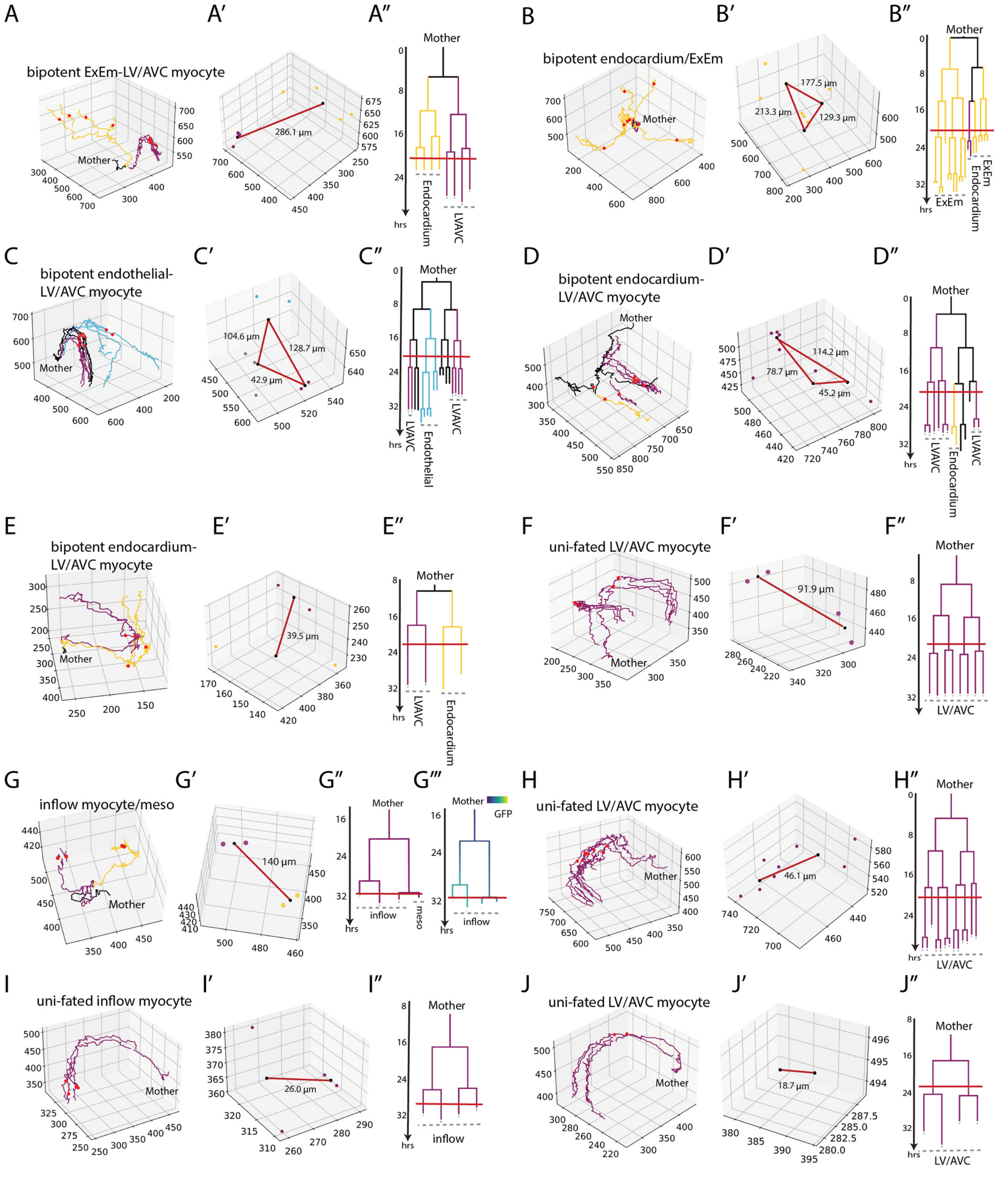

Figure EV4. Mesodermal migration paths.

Examples of trajectories (**A–J**) and corresponding midpoint distances (**A′–J′**) and lineage trees (**A″–J″**). Red lines mark sampling points for midpoint analysis. LV/AVC left ventricle and atrioventricular canal, ExEm Extraembryonic mesoderm. Source data are available online for this figure.

 © The Author(s)

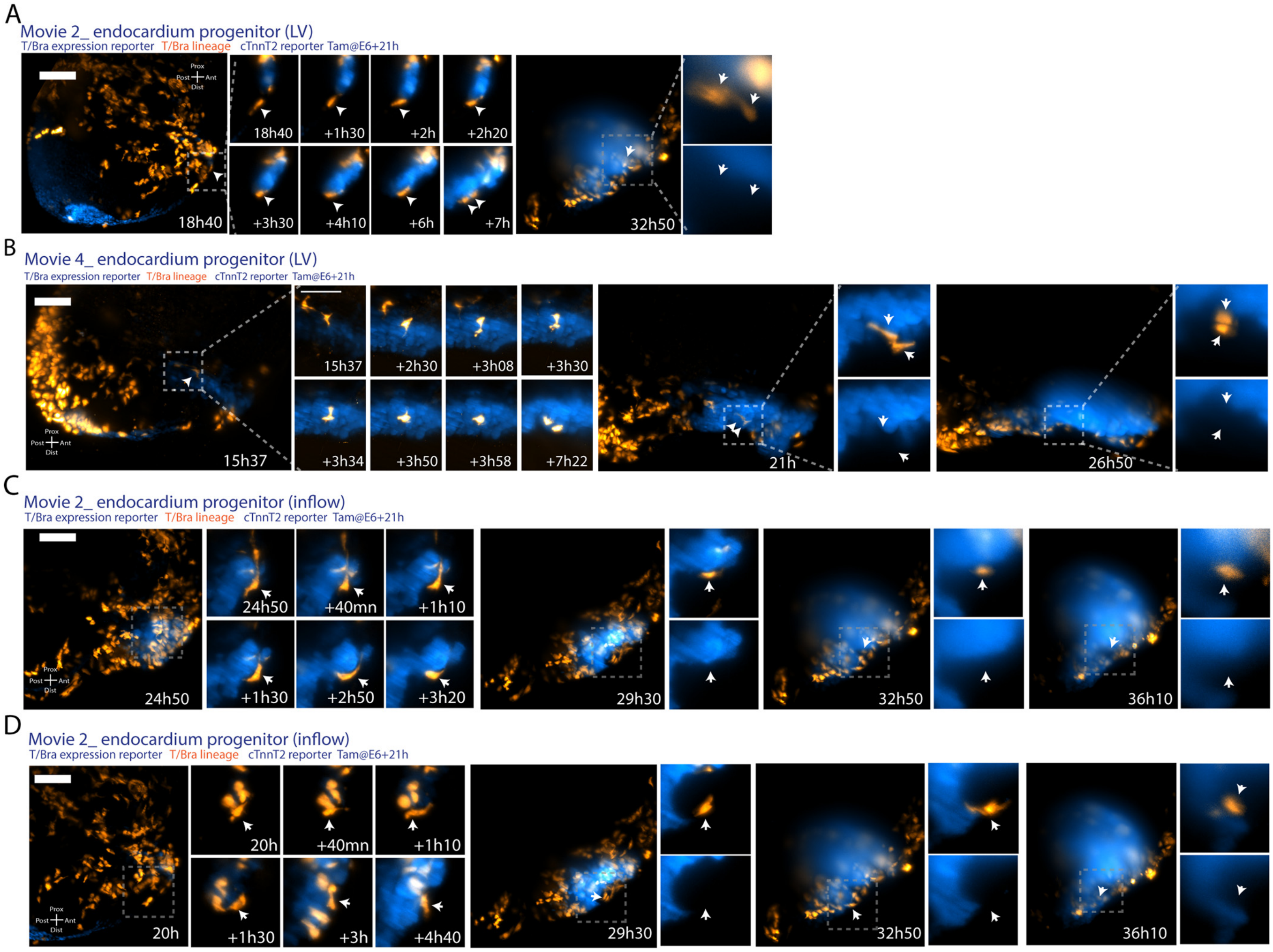

Figure EV5. Endocardial cell behaviour.

(**A–D**) Image sequences from time-lapse movies of *T*$^{nGPF\text{-}CreERT2/+}$;*R26R*$^{tdTomato/+}$; *cTnnT-2a-eGFP* embryos showing endocardial progenitors in the LV (**A**, **B**) and in the inflows (**C**, **D**). White arrows in (**A–D**) show endocardial cells. LV left ventricle. Scale bar: 100 µm.

© The Author(s)
